# Upper Critical Solution Temperature Polymer Phase Transition as a Tool for the Control of Inorganic Salt Crystallization Process

**DOI:** 10.3390/ma14185373

**Published:** 2021-09-17

**Authors:** Marcin Lemanowicz, Esteban Wong Munoz, Anna Mielańczyk, Krzysztof Kiraga, Andrzej Gierczycki

**Affiliations:** 1 Department of Chemical Engineering and Process Design, Faculty of Chemistry, Silesian University of Technology, ul. ks. M. Strzody 7, 44-100 Gliwice, Poland; Krzysztof.Kiraga@polsl.pl (K.K.); Andrzej.Gierczycki@polsl.pl (A.G.); 2 LOSENTECH SP. Z O.O., ul. Chabrowa 1, 44-210 Rybnik, Poland; esteban.wong@losentech.com; 3 Department of Physical Chemistry and Technology of Polymers, Faculty of Chemistry, Silesian University of Technology, ul. ks. M. Strzody 9, 44-100 Gliwice, Poland

**Keywords:** nucleation, phase transition, upper critical solution temperature (UCST), poly(acrylic acid) (PAA), KCl

## Abstract

In this paper, the experimental research concerning the impact of the hydrophilic-hydrophobic transition of a polymer exhibiting the Upper Critical Solution Temperature (UCST) onto the crystallization process of inorganic salt is presented. A hypothesis was postulated that under favorable process conditions the sudden change of macromolecules properties and the resulting appearance of insoluble particles will induce the nucleation process of the salt. Since the transition point parameters may be precisely designed, the described mechanism would eliminate the stochastic nature of the crystallization process. Although performed experiments proved that the postulated process mechanism was incorrect, the presence of macromolecules had a significant impact on the crystallization course. The stochastic nature of the process was not eliminated; nevertheless, it seems that a specific point of nucleation was created which was independent of the cloud point temperature (T_CP_) of the polymer. Moreover, the surface morphology of crystals was changed.

## 1. Introduction

The term “crystal engineering” was introduced by Schmidt in 1971 [1,2,3] and as a part of Chemical and Process Engineering plays a crucial role in the modern industrial technologies. Already at the beginning of the 21st century, 70% of all solid materials produced by the chemical industry were obtained via crystallization or precipitation [4]. At the present moment, one of the most important yet still unsolved issues in crystallization is to develop a precise control method to achieve designed product properties, such as crystal size and shape. One of the best examples is the manufacturing of pharmaceutical compounds. These processes are usually realized in batch reactors. On one hand, such an approach offers flexibility but on the other hand, it can cause problems of an economical nature due to the risk of obtaining off-specification products [5,6,7]. Another issue is the change of crystallizer scale, which is not as straightforward as it could appear [8], or the phenomenon of incrustation [7,9] which generates noticeable operational costs, especially in the industrialized economies [10]. Therefore multiple countries have tried to introduce high-quality standards of production by releasing guidelines such as Current Good Manufacturing Practice (CGMP) (Food and Drug Administration (FDA), USA) [11] or Best Available Techniques (BAT) Reference Documents (BREF) (European Integrated Pollution Prevention and Control (IPPC) Bureau (EIPPCB), EU) [12] which promote the development and implementation of advanced and, as a result, more efficient technologies.

One of the tools allowing for monitoring and control of the crystallization process is Process Analytical Technologies (PAT) which with appropriate feedback control technologies may raise the efficiency of production both in terms of economic performance and controllability of the products’ physical properties [5] by the employment of Quality by Design (QbD) and Quality by Control (QbC) philosophies [2]. This in turn minimizes the risk of crystal quality variation for different batches [13]. The PAT idea was introduced by the FDA in 2004 [2]. Yet still, multiple issues related to this approach have not been solved, e.g., monitoring of continuous processes or highly concentrated slurries parameters [2].

One of the key issues related to the isohydric crystallization by cooling is precise control of the nucleation phenomenon. In the case of uncontrolled nucleation, high supersaturation is generated and as a result dense population of small crystals is obtained. Therefore, it is crucial to know the Metastable Zone Width (MZW) to design the process properly. The MZW is defined as the difference between the saturation temperature of a solution and the temperature at which the first crystals appear during the continuous cooling process at a constant rate [14,15,16,17]. It is worth emphasizing that the crystallization phenomenon is very stochastic in nature [9,18,19,20] due to the simultaneous generation of a large number of nuclei. Therefore the MZW value is considered reproducible only under identical process parameters [15]. Furthermore, multiple factors influence the MZW. Among others, one can mention pH or the presence of impurities/additives (depending on whether the presence of the substance is intended or not, such as, for example, dissolved gases) [21,22,23]. Their impact on the crystallization phenomenon can be distinguished into two categories, i.e., thermodynamic effects (change of solubility) and kinetic effects (suppressing/accelerating crystal growth) [21].

**Figure 1:**
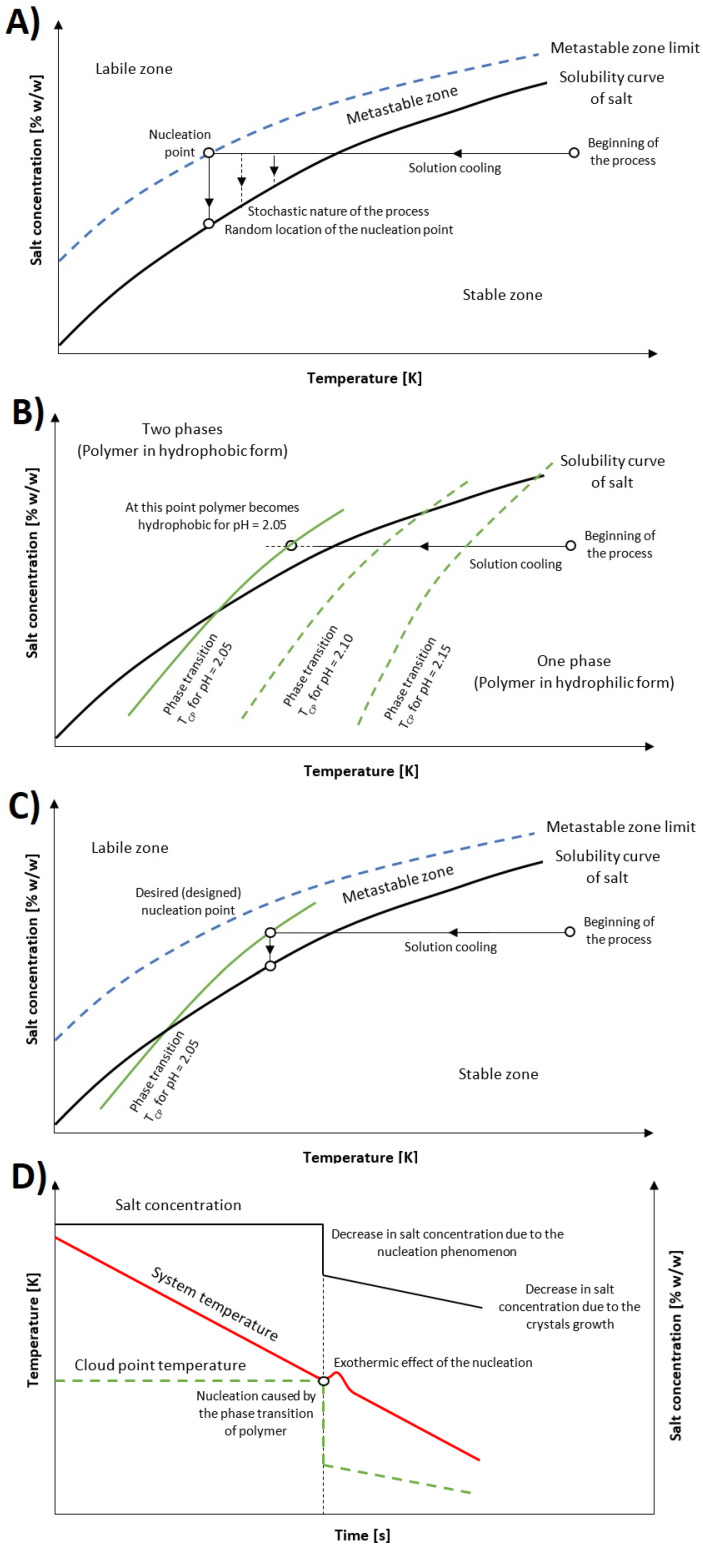
Proposed mechanism of crystallization: (**A**) standard crystallization; (**B**) phase transition of polymer, (**C**) combination of the processes, (**D**) process course in time.

In our opinion the seeding process is the weakest link in modern industrial crystallization. It introduces the risk of batch contamination and gives almost no control of crystal growth. Therefore, we have proposed a new approach to isohydric crystallization by solution cooling (i.e., for substances in which solubility increases with the raise in temperature in a significant way) that would allow one for the total elimination of the stochastic nature of the process. The research hypothesis is presented in Figure 1. The idea is as follows. When the solution is cooled down the solubility of a given substance is decreasing which is depicted by the horizontal arrow in the concentration vs. temperature graph (please note that this research does not concern the revers solubility substances in case of which usually the effort is made to inhibit the nucleation process and prevent scaling phenomenon [24]). At a certain temperature, the system enters the metastable zone, i.e., the concentration of dissolved matter is higher than it would appear from the solubility curve. In this state, the nucleation phenomenon may occur at any temperature. It is a completely random process that strongly influences the subsequent route of crystallization. On one hand, if the nucleation occurs near the solubility curve a small number of crystals with large final size will be created, which is often an undesirable feature of the product. On the other hand, if the nucleation occurs near the labile zone a numerous population of crystals with small final size will be created. This in turn will make their further processing, such as filtration and drying, more difficult [14,25]. Therefore, the seeding process is usually used in the industry to achieve the nucleation point at the desired point of the metastable zone width (MZW). 

At the present moment, polymers as tools in crystallization are intensively researched [24]. They can be used for confined crystallization as specialized microreactors. Moreover, the polymeric surfaces may promote nucleation, whereas free macromolecules may inhibit this phenomenon or influence the crystal growth. We propose an alternative solution, namely the application of stimuli-responsive polymer phase transition. Stimuli-responsive polymers significantly change their properties, such as their hydrophilic/hydrophobic state, due to the conformation transformation caused by small changes in environmental stimuli in either a continuous or stepwise manner [26,27,28,29,30]. In the case of temperature-sensitive polymers exhibiting Upper Critical Solution Temperature (UCST), the macromolecules change from hydrophilic to hydrophobic during the cooling process when T_CP_ is achieved. In our opinion, such a rapid change in the solution state will throw the system off-balance leading to the nucleation of dissolved matter (i.e., KCl). In the literature, there are other examples of (macro)molecules that undergo phase transition, defined as the transformation from disorder to ordered phase [31,32]. However, our studies did not address that issue. It has to be emphasized that the T_CP_ of thermoresponsive polymers may be precisely designed. Its value is determined by multiple factors such as polymer properties (molecular structure, molecular weight, dispersity, etc.), type of dissolved solids, or cooling rate. Therefore, when the system will achieve the characteristic point (T_CP_, C_CP_) in the concentration vs. temperature coordinate system, which will be placed within the metastable zone, the nucleation will occur. Another advantage of the presented mechanism results from the fact that usually UCST type polymers exhibit sensitivity to more than one stimulus. In the presented research we have used poly(acrylic acid) (PAA), which is also extremely sensitive to pH and dissolved matter concentration. Therefore, after nucleation the subsequent crystal growth will decrease the concentration of dissolved matter and in the result the polymer will become hydrophilic once again. 

The postulated mechanism would entirely remove the stochastic nature of the process. The nucleation point would occur at a precisely designed moment of the process. Moreover, the reverse transition of the macromolecules would simplify the separation and purification process of the product. Such a mechanism could substitute the seeding method used in industrial crystallization. Since the polymer would be present in the solution from the beginning of the process, higher sterility of the batch would be achieved. Finally, the presence of macromolecules may influence the growth of the crystals which in turn may influence their shape, habit, or size distribution.

In order to confirm our research hypothesis, the following questions have to be answered:

1. Will the phase transition initialize the nucleation process?
2. Will the concentration drop due to the crystal growth be sufficient in order to reverse the phase transition?
3. How will the presence of macromolecules influence crystal growth?

In general, UCST polymers have attracted only a small fraction of attention devoted to their LCST (Lower Critical Solution Temperature) counterparts, most likely because the UCST transition is usually less drastic and strongly dependent on solution pH and salinity in the suspending media [33]. The UCST behavior relies on hydrogen bonding (HB-UCST polymers) or Coulomb interactions (C-UCST polymers) [33]. In the first case, one may mention polymers such as poly(vinylmethylether), modified poly(vinyl alcohol)-s, or poly(hydroxyethyl methacrylate), which show both LCST and UCST features [34,35]. Unfortunately, the UCST lies outside the 273.15 to 373.15 K region. On the other hand, C-UCST macromolecules, such as some zwitterionic polymers, exhibit UCST behavior exclusively in pure water and very low ionic strengths, while like-charged C-UCST polymers, which require bridging interactions, are polymeric ionic liquids or function only in the presence of specific multivalent counterions [36,37]. In the presented research the poly(acrylic acid) was used. PAA and its copolymers exhibit UCST only at very high ionic strength (e.g., >400 mM NaCl) or low pH (pH < 4) [33].

This polymer is commercially available in the form of a water solution. We have already investigated the behavior of this macromolecule in the previous publication [38]. Among others, we proved that the relation between salt concentration, solution pH, and T_CP_ is very complex. Yet it is possible to design the process parameters in such a way that the T_CP_ will occur beyond the solubility curve of the salt.

For the presented research, KCl solution in deionized water was chosen. The reason was that this inorganic salt has been commonly used for many years and therefore extensive data concerning its crystallization are available in the literature. Among others one can mention publications concerning the impact of additives [39,40,41,42,43,44], surface charge [41], solvents [45,46,47,48] or pH [49]. Moreover, the nucleation process of KCl is highly exothermic, which therefore can be detected by precise temperature measurements. The presence of KCl also triggers the phase transition of PAA [38] thus it was a perfect candidate for this research.

The authors of this research are aware that the crystallization phenomenon may be influenced by multiple factors like stirring speed, cooling rate, etc. Therefore, in order to purely understand the mechanism of the proposed process, it was decided to focus only on the concentration of salt, since it would change during the process course, and the pH of the solution, which directly controlled the value of the cloud point temperature. The rest of the parameters were constant in all measurements. 

## 2. Materials and Methods

In the experiments, KCl (VWR Chemicals, Gdańsk, Poland) solutions in deionized water (Hydrolab, Poland) were used. Poly(acrylic acid) (240,000 Da, 25% *w*/*w* water solution, analytical grade) was purchased from Acros Organics. The pH of solutions was adjusted by the addition of HCl (analytical grade).

Transmittance was measured using a specially designed apparatus comprised of an Agilent Cary 60 spectrometer (MS Spectrum, Warsaw, Poland) and a Quantum Northwest TC-1 temperature controller coupled with specialty software (MS Spectrum, Warsaw, Poland). Before each analysis, a quartz cuvette (10 mm optical path), 7 mm magnetic stirrer, and a Pasteur pipette were preheated in a laboratory dryer at least 10 K above the saturation temperature. The Peltier holder for the cuvette was also preheated. All samples were freshly prepared before measurements. Firstly 20 mL of primary sample was prepared at a temperature 10 K higher than the saturation temperature. Then 3.5 mL of solution was transferred using Pasteur pipette to the cuvette and instantaneously inserted to the Peltier holder. The sample was stabilized for 30 min. During the stabilization stage as well as the measurements the stirrer was working with 1000 RPM providing intense mixing and therefore homogenization of crystal suspension in the last stage of the experiments. This value was determined based on trial experiments during which the stability of the transmittance measurement was investigated. During experiments, three parameters were directly measured, namely sample temperature (using temperature sensor placed inside the cuvette), holder temperature, and transmittance. All measurements were performed with a 1 K/min cooling rate. This value is a compromise between the cooling rates expected for the crystallization process, values used for investigations of stimuli-responsive polymers, and the time needed to perform the measurement. Each sample was measured twice—this process was automated by the apparatus software. After the cooling cycle, the sample was mixed for 30 min at 288.15 K. Then it was heated to the temperature 10 K higher than the saturation temperature with a heating rate equal to 1 K/min and stabilized for another 30 min. At this point, the transmittance was measured once again during the cooling process. Each measurement point was investigated at least 5 times (i.e., at least 5 separate, identical samples were prepared). If the obtained results were not consistent, additional trials were performed.

After the crystallization process, the obtained crystals were recovered for further analysis. For this purpose, two strategies were employed. In the first case, the suspension was filtered using a funnel with sintered filter under low pressure and the crystals were washed with methanol (analytical grade) to remove the remaining solution and prevent further crystallization and resulting aggregation. However, since methanol would remove residuals of PAA, in the second case the washing step was omitted. Next, the crystals were dried in a laboratory dryer and analyzed using a CH30 Olympus optical microscope equipped with a light polarization add-on and a digital camera, and a Phenom ProX scanning electron microscope by Thermo Fisher Scientific equipped with EDS add-on.

At the beginning of the research, the process parameters have to be determined based on our previous experiments [38]. In order to eliminate too many unknowns from the research one fixed concentration of PAA was used in all experiments, equal to 0.25% *w*/*w*. Moreover, it was decided that three different KCl concentrations would be used, i.e., 27, 28, and 29% *w*/*w*, because corresponding saturation temperatures were in the range allowing for operation of laboratory glassware with bare hands and simultaneously the nucleation temperature was high enough that it was not necessary to wash the cuvette with dry air to prevent moisture condensation [38]. Three specific points in the concentration vs. temperature coordinate system corresponding to the T_CP_ were selected for each KCl concentration: below saturation curve (stable zone), within the metastable zone, above the metastable zone (labile zone). As was described earlier we intended to place the T_CP_ within the metastable zone to initialize the nucleation process. Two additional points allowed us to investigate the overall impact of polymer present in the hydrophilic and hydrophobic forms on the crystallization course. The points below metastable zone corresponded to pH = 2.10, within the metastable zone to pH = 2.05 and above the metastable zone to pH = 2.00. The pH was adjusted with +/− 0.01 accuracy. Concluding, a grid of 9 points was created. Their position was determined based on trials and error methods.

## 3. Results and Discussion

At the beginning of this research, a greater number of samples were prepared during one day. Part of these samples was investigated immediately after preparation whereas the rest was stored at 5 °C. However, it turned out that the results of stored samples deviated significantly from the results of fresh samples. Similar observations were made by Pineda-Contreras and coworkers [50]. In their work, the stability of acrylamide and acrylonitrile UCST-type copolymers was investigated. The samples’ T_CP_ was measured up to 25 days after synthesis in different conditions. They proved, among others, that for specific conditions the thermosensitivity of macromolecules can disappear after such a long period of storage time. They reached conclusions, with which we fully agree, that some irreversible changes of the macromolecule’s chemical structure occurred due to the harsh, acidic conditions and high concentration of salt (which ions interactions with the polymer is the key condition of thermosensitivity of PAA). Moreover, at this point, it has to be emphasized that the PAA was extremely sensitive to the pH change. Even a very small addition of 0.1 M HCl changed the T_CP_ value significantly. Since the samples had to be heated to the temperature above the saturation temperature after the storage, the delicate balance of components could be destroyed.

**Figure 2:**
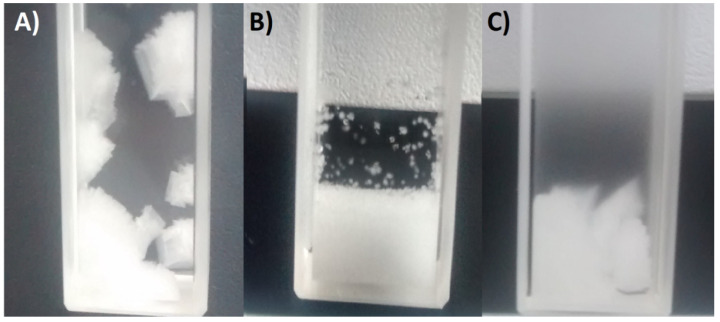
KCL crystals from 30% *w*/*w* solution: (**A**) Solution without any additives, no mixing during cooling; (**B**) solution without any additives, 1000 RPM mixing; (**C**) solution with 0.25% *w*/*w* PAA, no mixing during cooling.

**Figure 3:**
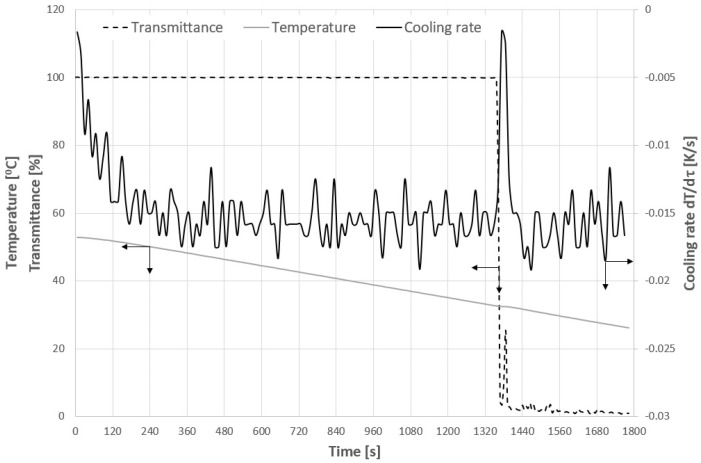
Exemplary measurement results for 29% *w*/*w* KCL solution.

Figure 2 presents the photographs of crystals from the initial stage of the research. When the crystallization was performed without any mixing large, aggregated crystals were obtained, often attached to the walls of the cuvette (Figure 2A). In order to prevent a situation in which the optical path would not be obscured by the crystals, we have used a 7 mm magnetic stirrer placed within the vessel. The mixing speed was established based on the stability of the absorbance readings (Figure 3) when crystals were present in the sample; 1000 RPM mixing provided full homogenization of the suspension. Of course, as one could expect, the hydrodynamic conditions had to affect the crystal size distribution. Significantly smaller particles were created. However, after careful examination, one can notice that still small part of the crystals was attached to the walls of the cuvette (Figure 2B). The situation changed when the samples with PAA addition were crystallized (Figure 2C). As in the case of pure KCl solution without mixing large crystals were obtained. Yet it needs to be emphasized that none of them were attached to the walls of the cuvette. Additionally, Figure 2C demonstrates the turbidity of the solution resulting from the presence of hydrophobic particles of PAA. As it was proven in our previous research [38], PAA macromolecules are extremely sensitive to the change of pH. In order to fit the T_CP_ for a given salt concentration, the pH value has to be adjusted.

The incrustation/scale inhibiting properties of PAA are already known. For example, Zhang and coworkers [51] compared the performance of modified scale inhibitors as well as commercially available products for calcium sulfate. Among others, they proved that the efficiency of a commercial product JH-907, which is a commonly used polyacrylic acid derivative inhibitor, achieved 80%. Similar results were obtained by Popov and coworkers [52] for commercial poly(acrylic acid) sodium salt, whereas Du and coworkers [53] reported dual functionality of poly(acrylic acid) grafted starch by terms of scale inhibition and suspension destabilization (flocculation). In all of the abovementioned cases, authors indicate the same process mechanism, namely the interaction between the cation of the salt and the functional groups of polymers. Additionally, in the last case authors indicate that the effects of dispersion or crystal lattice distortion due to the distinct linear branched-chain structure and amphiphilic features of the polymer may indirectly improve the scale inhibition efficiency for already formed crystals. These facts imply interesting conclusions concerning our work. The presence of PAA in the solution should influence the solubility of the KCl salt. Moreover, since during the nucleation process the phase transition of polymer is not instantaneous, i.e., the whole population of macromolecules will not change their properties from hydrophilic to hydrophobic at the same moment; both forms of PAA should inhibit the formation of the crystalline matter at the heat exchange surfaces. That would be a significant improvement of the crystallization process since the annual cost due to all types of fouling in industrialized countries is estimated at the level of 0.25% of their gross domestic product (GDP), which gives about 35.75 billion euro for the European Union in 2014 [9,54]. Once again we would like to emphasize that according to our research hypothesis at the end of the crystallization process, whole PAA would be hydrophilic. Therefore, it should be removed with the liquid whereas the crystalline product should be pure.

Figure 3 presents the exemplary measurements of the crystallization process at the initial stage of the research. During the cooling process the temperature of the sample and the cuvette holder, as well as the transmittance of the sample, were measured. The rise of temperature during nucleation, which is an exothermic process, was relatively small. Since we wanted to eliminate the “human factor” for the assessment of nucleation point it was determined based on the cooling rate value which was calculated as the numerical time derivative of temperature. Of course, as one should expect due to the intense mixing, the nucleation also resulted in a sudden transmittance drop. This graph is presented to demonstrate the method of distinguishing between the transmittance drop due to the nucleation and due to the polymer transition. In the second case, the change was not accompanied by the change in the cooling rate. Therefore, the nucleation point could be precisely determined even in the middle of polymer phase transition. It is also worth mentioning that our previous research [38,55] proved that the phase transition of PAA is less drastic in comparison to the sudden drop of transmittance in the case of LCST type polymers. It will be visible in the discussion of the following results. At this point, the authors would like to strongly emphasize that although the crystallization was realized within a cuvette placed in a spectrometer the process was investigated using the commonly acknowledged polythermal method. The transmittance measurements were necessary in order to detect phase transition of polymer. It is also worth noticing that the presented research fits well with the idea of PAT, i.e., analysis of the phenomenon using multiple measurement techniques. The nucleation point could be detected using two independent stimuli. In all experiments a small time delay was noticed between the change of transmittance and temperature. It resulted from several reasons. Firstly, to detect the temperature rise in sample the temperature of the sensor had to be equalized with a temperature of suspension. This process was significantly slower compared with the measurement of light transmittance. Secondly, to determine the time derivative of temperature, a numerical method was used in which given time step had to be used. Simultaneously one has to remember that the temperature change occurred within the whole volume of the sample, whereas to detect the transmittance change the crystals had to appear directly in the path of the light.

**Figure 4:**
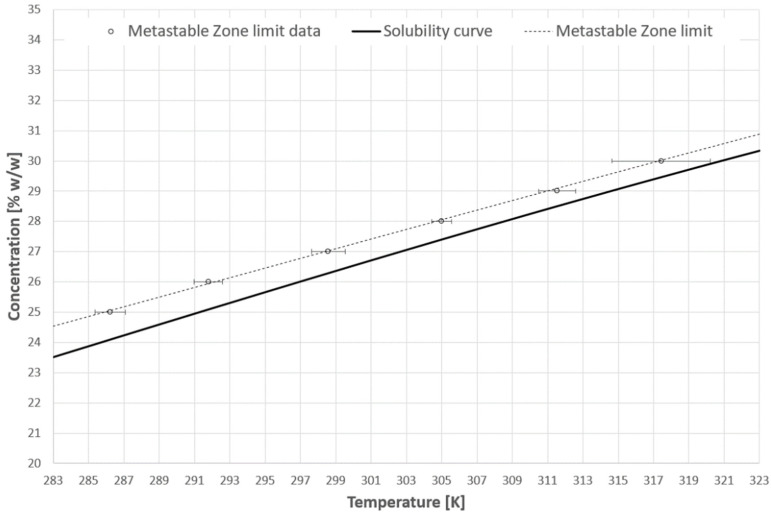
Metastable zone.

The first task was to determine the metastable zone width using the polythermal method. The results are presented in Figure 4. The solubility curve was taken from the literature [56]. It is represented by the following equation:

(1) $$S=10^{A+B\cdot T+C\cdot T^{2}},$$

where:

*A* = −0.701174631495441; *B* = 1.13176341377697 × 10^−2^; *C* = −1.41129148146649 × 10^−5^

The metastable zone limit was presented by us using the same equation (Equation (1)). In this case, the coefficients took the values:

*A* = −0.058768988492580; *B* = 0.741557163318996 × 10^−2^; *C* = −0.811467496530891 × 10^−5^

The coefficient of determination for this function was equal to 0.997. The mean, as well as the maximum values of supercooling, are presented in Table 1.

The mean value of the pH of the initial solution was equal to 6.51 (std. dev. 0.21). The slight acidity pH value of the samples resulted from the absorption of gases from the atmosphere by deionized water. Generally speaking, one may notice that the higher the concentration of the salt, the narrower the metastable zone width. This tendency is disturbed by the results for the 30% solution. In this case, the highest dispersion of measured temperature was noticed. It resulted from the fact that the volume of the samples was very small, whereas their temperature had to be high compared to the rest of the experiments at this stage, which promoted evaporation of water. Since the solution in a preheated cuvette temperature was approximately 333 K, it was difficult to instantaneously transfer the liquid from the batch sample to the spectrometer.

**Figure 5:**
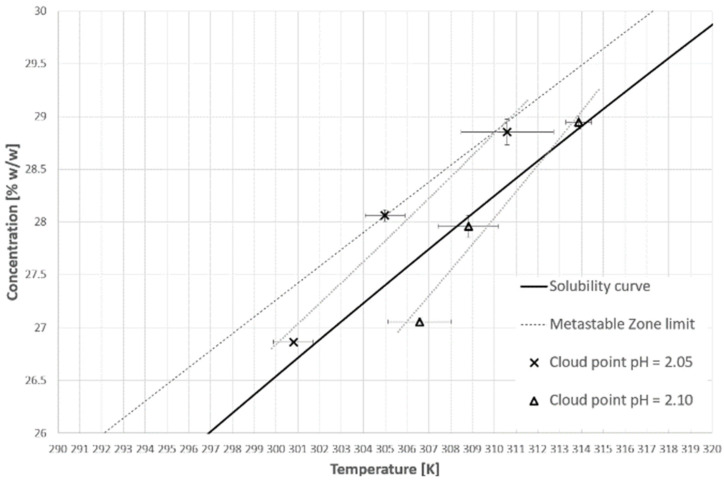
Cloud point temperatures for the first cooling cycle.

**Figure 6:**
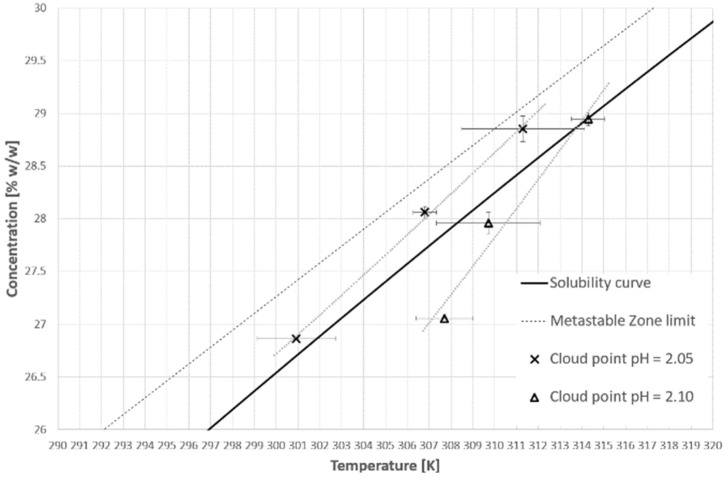
Cloud point temperatures for the second cooling cycle.

Figure 5 and Figure 6 represent the cloud points of PAA for the first and second cooling cycles. The dot lines are used only for the visualization of the limit between the hydrophobic and hydrophilic states of the polymer. Firstly, it has to be emphasized that the T_CP_ was determined based on the first noticeable change of transmittance, in contrast to the method which was used by us in the previous publication [38] based on the analysis of the second temperature derivative of transmittance. This approach results strictly from the research hypothesis. In our opinion, the appearance of the first hydrophobic macromolecules should initialize the nucleation process. Therefore, it is pointless to use the inflection point of the transmittance since it appears more or less in the middle of the population transition phenomenon. Secondly, we would like to draw the reader’s attention to our previous results [38]. Although the trend lines suggest that the cloud point will move below the solubility curve, it will not. We already proved that for each salt concentration one can find a limiting pH value below which the transition will not occur.

We expected that some dispersity of cloud point temperatures would be inevitable due to the complexity of interactions between components of the samples. It was higher than in our previous research [38] (in which the repeatability was excellent), yet it is comparable with the results of other authors [57]. Due to the presence of crystals within the samples, it was impossible to analyze the hysteresis between the cooling and heating cycles as it is often encountered in other papers dedicated to the UCST polymers [58]. However, the thermal history of the solution had an impact on the phase transition. To our surprise, when two consecutive crystallizations were realized using the same sample, the cloud point of the polymer solution during the second cooling stage was usually higher than during the first cooling stage. On one hand, similar results were presented by Seuring and Agarwal [59]. However, in their case, the T_CP_ of poly(*N*-acryloylglycinamide-*co*-butyl acrylate) decreased with each of nine consecutive runs. As in the case of the sample storage issue discussed earlier in this paper, this phenomenon is associated with the irreversible changes of polymer chemical structure. On the other hand authors of [60,61] proved that the thermal history of the samples had no greater influence on the value of T_CP_ for UCST transition of poly(ethylene glycol) and poly(acrylamide-*co*-acrylonitrile) copolymers and poly(*N*-acryloyl glycinamide), respectively. The results presented in the latter paper are especially interesting from our point of view because the samples were stored at 6.3 °C for 1 h before measurements. Concluding, in our opinion, when the polymer achieved the hydrophobic state during the cooling process, the extreme conditions (low pH, high salinity) irreversibly changed its chemical structure. Therefore, the course of the second cooling stage or the course of the cooling stage of a previously stored sample was altered.

**Figure 7:**
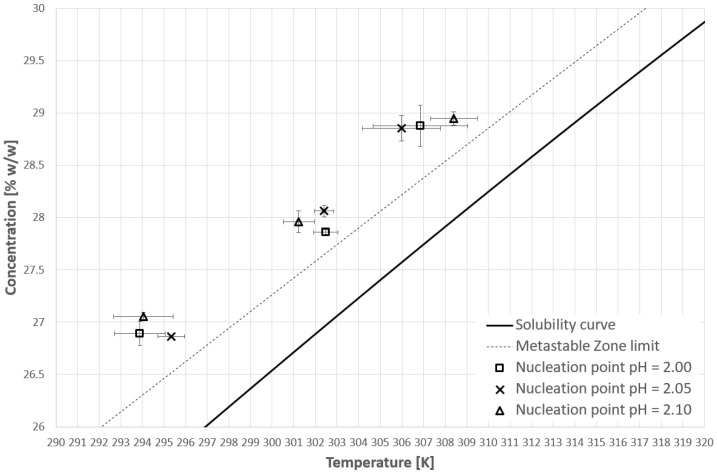
Nucleation point temperatures for the first cooling cycle.

**Figure 8:**
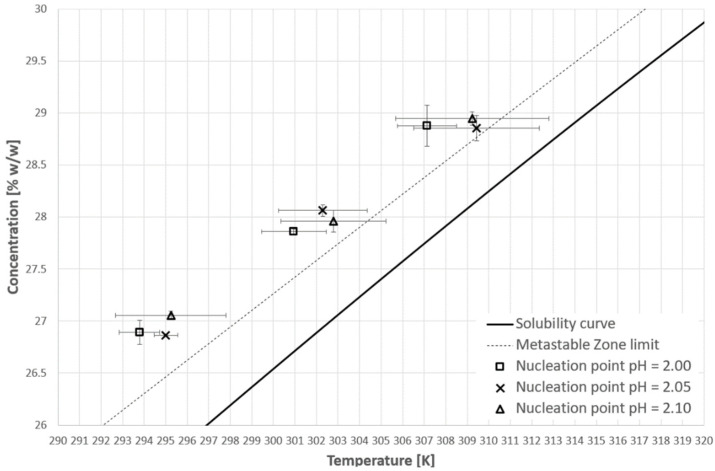
Nucleation point temperatures for the second cooling cycle.

Figure 7 and Figure 8 represent the nucleation points for the first and second cooling cycle, respectively. Unfortunately, these results completely disprove our research hypothesis. As one may notice in all cases (T_CP_ below solubility curve—pH = 2.10; T_CP_ within the metastable zone—pH = 2.05; no phase transition—pH = 2.00) the nucleation points are well beyond the metastable zone determined for the pure KCl solution. It seems that the state of macromolecules had no greater impact on that phenomenon. What is more, the alteration of the process negatively influences its randomness. The dispersity of the results was higher compared to the pure solution, especially in the case of the second cooling cycle. What is interesting is that the maximal value of supercooling is noticeably higher compared to the initial results (Table 1 and Table 2).

The impact of impurities and admixtures onto the crystallization process has been investigated for many years [62,63], also in the case of potassium chloride [41,42,45,46]. For example, the impact of the pH value on the growth and dissolution rate of potassium chloride was investigated in [49] by Mohameed and Ulrich. They proved, among others, that lower pH promotes these phenomena comparing to neutral pH, resulting in higher growth and dissolution rate depending on the supersaturation value. Whereas Ceyhan and Bulutcu [41] suggest that these processes are also affected by the surface charge of the crystals. Although we could not find any report concerning the impact of the pH value on the MZW of KCl, the paper of Al-Jibbouri and coworkers delivers some interesting suggestions on this topic. They investigated the crystallization kinetics of epsomite. Unlike the work of Mohameed and Ulrich, they proved that lowering the pH value resulted in decrease in growth and dissolution rates. Moreover, the research showed that the raise in pH value narrowed the MZW whereas lower pHs did not influence this system property.

**Figure 9:**
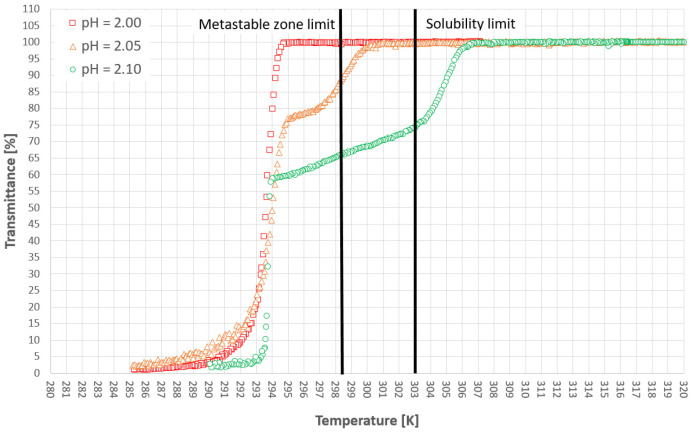
Process course for 27% *w*/*w* KCl solution.

**Figure 10:**
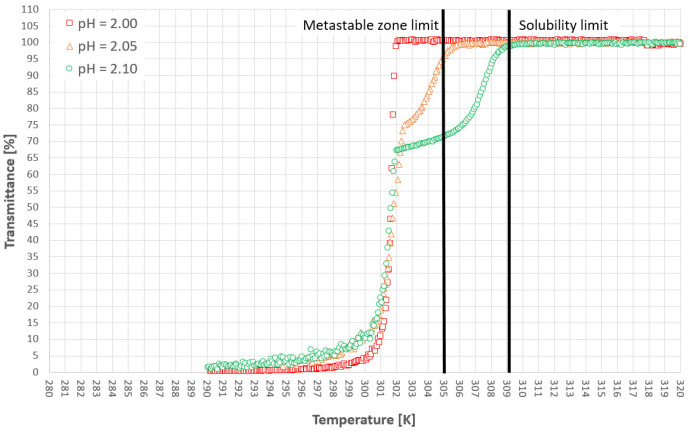
Process course for 28% *w*/*w* KCl solution.

**Figure 11:**
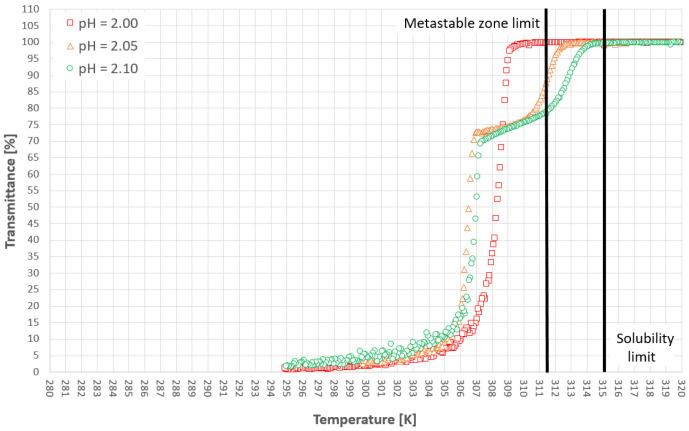
Process course for 29% *w*/*w* KCl solution.

Figure 9, Figure 10 and Figure 11 present selected results from our experiments for KCl concentration equal to 27, 28, and 29% *w*/*w,* respectively, for the first cooling cycle. We would like to emphasize that these data were selected from a significant number of measurements which were characterized by noticeable dispersity of points as shown in Figure 7. However, we would like to depict an interesting behavior of the system. It was noticed that for each concentration one could easily find the set of process courses for which the nucleation point appeared at a very similar temperature. Several conclusions may be made from this observation. Firstly, the state of macromolecules (hydrophilicity/hydrophobicity) has no impact on the process. It seems that the hydrophobic particles of PAA are invisible from the nucleation point of view. Moreover, the very fact of the phase transition did not destabilize the system as it was assumed in the presented research hypothesis. The nucleation point appeared for a specific temperature, no matter how fast the cloud point occurred during the cooling of the solution. Secondly, in the investigated range of pH values, the acidity of the solution had no clear impact on the MZW. As one may notice in Figure 7, Figure 8, Figure 9, Figure 10 and Figure 11, the ranges of results for each pH overlap. The mechanism of this phenomenon is not clear. For example, if the macromolecules of PAA would inhibit the creation of the crystal nucleus then the point of phase transition had to influence the time needed for nucleation. This was noticed in the presented research. On the other hand, the interaction between KCl ions and PAA macromolecules is the basic condition of the polymer’s thermosensitivity. Perhaps, the action of PAA is bidirectional? This means that the phase transition of polymer initializes the formation of crystals nucleus; however, their growth is hampered or stopped by the formation of a buffer layer composed of polymer chains. Therefore, until a critical supercooling is not reached no crystal entities may be created.

**Figure 12:**
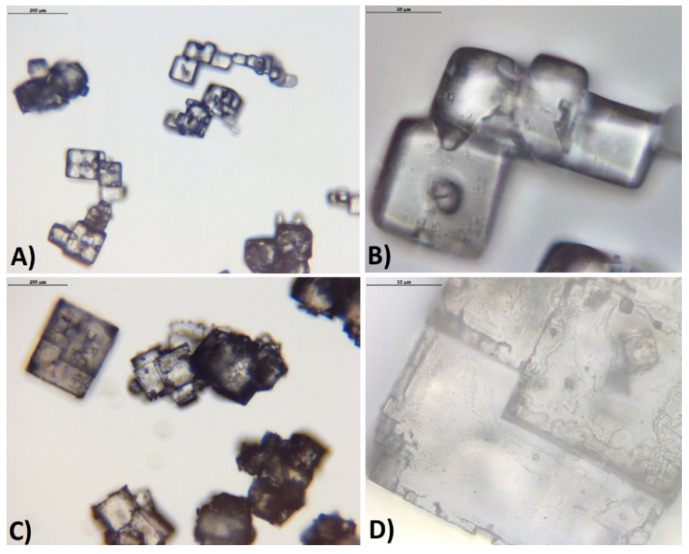
Optical microscope photographs of KCl crystals: (**A**) 29% *w*/*w* pure solution (200 m length scale); (**B**) 29% *w*/*w* pure solution (50 m length scale); (**C**) 29% *w*/*w* solution with PAA pH = 2.10 (200 m length scale); (**D**) 28% *w*/*w* solution with PAA pH = 2.10 (50 m length scale).

**Figure 13:**
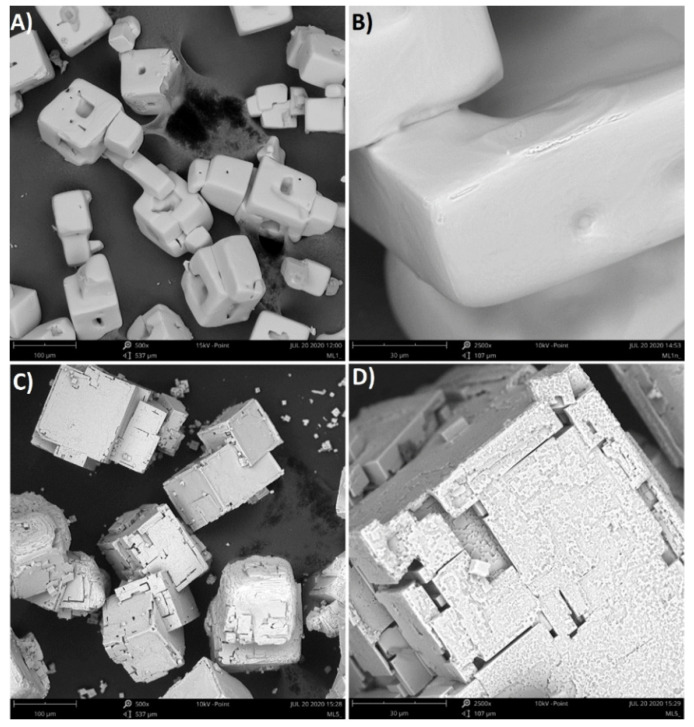
SEM photographs of KCl crystals: (**A**) 29% *w*/*w* pure solution (200 m length scale); (**B**) 29% *w*/*w* pure solution (50 m length scale); (**C**) 29% *w*/*w* solution with PAA pH = 2.10 (200 m length scale); (**D**) 29% *w*/*w* solution with PAA pH = 2.10 (50 m length scale).

This mechanism proposal may be supported by the videos of crystals growth (see Supplementary Materials) and photographs of crystals obtained using pure KCl solution and solution with the addition of PAA (Figure 12 and Figure 13). In the first case, hopper-cubic crystals were noticed with relatively smooth faces and rounded corners. Identical crystals were obtained, for example, in [44,48]. The cavities at the center of the walls may be attributed to mother liquor occlusion whereas the edge roundness resulted from mechanical attrition. However, if the polymer was present in the solution the crystal habit changed noticeably. The crystal had a cubic shape, however in this case no holes at the center of the walls were present. The edges of the crystals were very sharp. This observation proves that the presence of polymer influenced the growth of the crystals. In our opinion, PAA macromolecules provided a buffer layer between the crystal and the solution which ensured steady crystal growth (mass transfer between mother liquor and solid body) and protection from mechanical damage.

Crystals that in part resemble those received by us were presented by Rohani and Ng [44]. They were created in the presence of silica powder. Authors concluded that small, insoluble particles promoted the formation of rough surfaces. In order to investigate this phenomenon further, the growth of crystals in both cases was recorded. They are attached as Supplementary Materials to this paper. In the case of a pure solution, one may notice that growth was towards the diagonals of the crystal. The center of each wall remained empty for a long time. The crystalline matter filled the cavities at the later stages of crystal growth which resulted in the hopper-cubic shape. However, in the second case, the growth of each wall was smooth and uniform which led to the formation of sharp-edged crystals.

**Figure 14:**
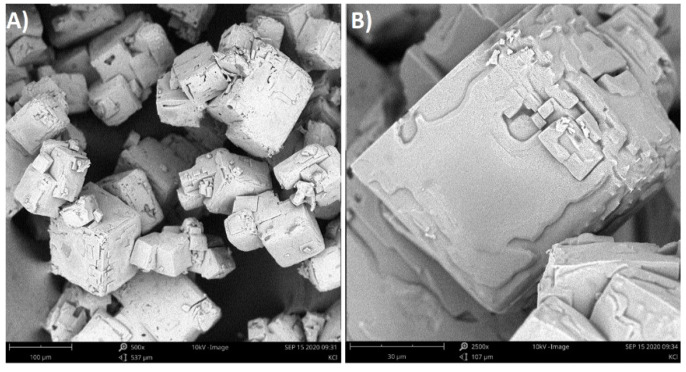
SEM photographs of KCl crystals without methanol rinse: (**A**) 29% *w*/*w* solution with PAA pH = 2.10 (200 m length scale); (**B**) 29% *w*/*w* solution with PAA pH = 2.10 (50 μm length scale).

In this situation a new question arises: will the macromolecules incorporate into the crystal structure? In order to answer this question, energy-dispersive X-ray spectroscopy was used. However, all crystals surfaces were composed of K and Cl elements only. Since the crystals were rinsed with methanol directly after filtration, a possibility existed that the PAA macromolecules could be washed away. Therefore, the additional SEM photographs of crystals that were just dried in a laboratory dryer were taken (Figure 14).

**Figure 15:**
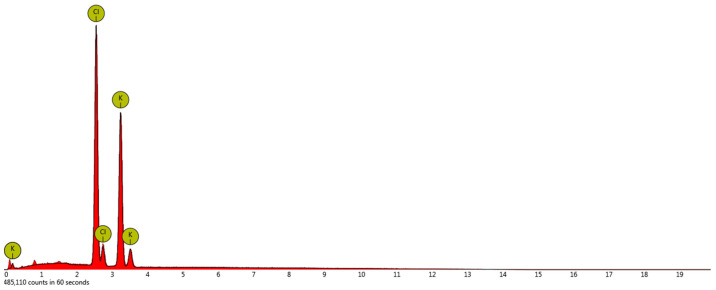
EDS analysis of the crystals surface (crystallization of 29% *w*/*w* solution, pH = 2.10 with PAA).

The obtained crystals had more smooth surfaces. Moreover, in most cases, they were aggregated. These results should be expected since the mother liquor was not removed, leading to further crystallization and aggregation. However, the re-analysis of surface composition did not reveal any changes comparing to the previous results (Figure 15). The crystals surfaces were composed only of K and Cl elements.

## 4. Conclusions

In this paper, a new mechanism of crystallization was proposed. The research hypothesis was based on the employment of the phase transition phenomena of UCST-type polymer. The sudden appearance of hydrophobic particles should destabilize the solution leading to controlled nucleation. Unfortunately, the experimental research proved otherwise. Based on our work the following conclusions may be made:

1. The application of PAA resulted in the reduction in the incrustation process. This fact may greatly reduce the operating costs of industrial crystallization plants;
2. The application of PAA and lowering the pH of the solution broadened the MZW. Both meant supercooling and maximal supercooling was noticeably increased;
3. The phase transition of PAA had no impact on the nucleation process. It seems that the position of cloud point temperature (below the solubility curve, within the metastable zone, no cloud point) did not influence the supercooling values;
4. Although the stochasticity of the crystallization phenomenon was not reduced, a specific value of supercooling could be found which was common for all investigated values of pH. In our opinion this fact may be explained by the bidirectional action of PAA macromolecules: on one hand, the formation of crystal nucleus was promoted, but on the other hand, the nucleus was shielded from further growth;
5. The presence of PAA significantly influences crystal growth and habit. As in the previous point, the phenomenon may be associated with the presence of a polymer buffer layer between the KCl crystal and the supersaturated solution.

## Figures and Tables

**Table 1:** Metastable zone width.

	Concentration of KCl [% *w*/*w*]					
	25	26	27	28	29	30
ΔT_MEAN_ [K]	4.93	5.36	4.07	3.64	3.09	3.21
Std. dev. [K]	0.85	0.80	0.96	0.55	1.05	2.77
ΔT_MAX_ [K]	6.58	6.73	5.45	3.86	4.28	7.24

**Table 2:** Metastable zone width in the presence of PAA (values in brackets represent standard deviation).

Concentration of KCl [% *w*/*w*]	26.89 (0.11)	26.87 (0.01)	27.06 (0.04)	27.86 (0.03)	28.06 (0.06)	27.96 (0.10)	28.88 (0.20)	28.86 (0.12)	28.95 (0.06)
pH	2.00	2.05	2.10	2.00	2.05	2.10	2.00	2.05	2.10
ΔT_MEAN_ [K] (first cooling cycle)	8.27 (1.17)	6.82 (0.61)	8.61 (1.37)	5.67 (0.57)	6.74 (0.44)	7.42 (0.71)	7.31 (2.60)	8.17 (1.81)	5.75 (1.08)
ΔT_MAX_ [K] (first cooling cycle)	9.61	7.25	9.75	6.20	7.40	8.06	9.46	10.27	6.99
ΔT_MEAN_ [K] (second cooling cycle)	8.36 (0.94)	7.14 (0.55)	7.41 (2.56)	7.20 (1.50)	6.86 (2.07)	5.85 (2.44)	7.01 (1.36)	4.71 (2.92)	4.92 (4.25)
ΔT_MAX_ [K] (second cooling cycle)	9.25	7.19	10.37	8.45	8.83	7.32	8.25	8.85	9.55

## Supplementary Materials

The following are available online at https://www.mdpi.com/article/10.3390/ma14185373/s1, Video S1: Pure_KCL, Video S2: KCl_and_PAA.
